# Age-Related Adaptations of Lower Limb Intersegmental Coordination During Walking

**DOI:** 10.3389/fbioe.2019.00173

**Published:** 2019-07-17

**Authors:** Mathieu Gueugnon, Paul J. Stapley, Anais Gouteron, Cécile Lecland, Claire Morisset, Jean-Marie Casillas, Paul Ornetti, Davy Laroche

**Affiliations:** ^1^INSERM, CIC 1432, Module Plurithematique, Plateforme d'Investigation Technologique, Dijon, France; ^2^CHU Dijon-Bourgogne, Centre d'Investigation Clinique, Module Plurithématique, Plateforme d'Investigation Technologique, Dijon, France; ^3^Neural Control of Movement Laboratory, Faculty of Science, Medicine and Health, School of Medicine, University of Wollongong, Wollongong, NSW, Australia; ^4^INSERM, UMR 1093-CAPS, Université de Bourgogne Franche Comté, UFR des Sciences du sport, Dijon, France; ^5^Department of Physical Medicine and Rehabilitation, Dijon-Bourgogne University Hospital, Dijon, France; ^6^Damartex Group, Roubaix, France; ^7^Department of Rheumatology, Dijon University Hospital, Dijon, France

**Keywords:** gait analysis, aging, panar covariation, biomechanics, locomotor control

## Abstract

Lower-limb intersegmental coordination is a complex component of human walking. Aging may result in impairments of motor control and coordination contributing to the decline in mobility inducing loss of autonomy. Investigating intersegmental coordination could therefore provide insights into age-related changes in neuromuscular control of gait. However, it is unknown whether the age-related declines in gait performance relates to intersegmental coordination. The aim of this study was to evaluate the impact of aging on the coordination of lower limb kinematics and kinetics during walking at a conformable speed. We then assessed the body kinematics and kinetics from gait analyses of 84 volunteers from 25 to 85 years old when walking was performed at their self-selected speeds. Principal Component Analysis (PCA) was used to assess lower-limb intersegmental coordination and to evaluate the planar covariation of the Shank-Thigh and Foot-Shank segments. Ankle and knee stiffness were also estimated. Age-related effects on planar covariation parameters was evaluated using multiple linear regressions (i.e., without a priori age group determination) adjusted to normalized self-selected gait velocity. Colinearity between parameters was assessed using a variation inflation factor (VIF) and those with a VIF < 5 were entered in the analysis. Normalized gait velocity significantly decreased with aging (*r* = −0.24; *P* = 0.028). Planar covariation of inter-segmental coordination was consistent across age (99.3 ± 0.24% of explained variance of PCA). Significant relationships were found between age and intersegmental foot-shank coordination, range of motion of the ankle, maximal power of the knee, and the ankle. Lower-limb coordination was modified with age, particularly the coordination between foot, and shank. Such modifications may influence the ankle motion and thus, ankle power. This observation may explain the decrease in the ankle plantar flexor strength mainly reported in the literature. We therefore hypothesize that this modification of coordination constitutes a neuromuscular adaptation of gait control accompanying a loss of ankle strength and amplitude by increasing the knee power in order to maintain gait efficiency. We propose that foot-shank coordination might represent a valid outcome measure to estimate the efficacy of rehabilitative strategies and to evaluate their efficiency in restoring lower-limb synergies during walking.

## Introduction

Human walking is a common task with efficient motor control. Synergic muscle activation for the control of limb movements requires the integration of inputs from the central nervous system and feedback from proprioceptive sensors in the muscles, tendons, and limbs. In healthy persons, the neural command ensures a rhythmic, stable gait with a highly consistent intersegmental coordination, and overall walking patterns. This coordination, corresponding to the process of mastering redundant degrees of freedom of the body into a controllable system, allows the efficiency of gait by maintaining dynamic equilibrium, and the lowest energetic cost during gait (Bernstein, 1967; Lacquaniti et al., 1999). The movement coordination during gait might therefore reflect neuro-muscular synergies. While an inability to modulate the intersegmental coordination may induce gait deviations, it might also provide insights into the organization and adaptation of gait patterns with pathology or aging (Winter et al., 1990).

Declining mobility and gait performance is one of the major functional hallmarks of aging (Boyer et al., 2017). Age-related differences in gait performance include a decrease in gait speed, a reduction in step length, and/or an increased cadence (Mcgibbon and Krebs, 2001; Lewis and Ferris, 2008). These changes are associated with impaired balance control, a reduction of muscle strength, and mass as well as an increase of the energy cost of walking (Sepic et al., 1986; Winter et al., 1990; Judge et al., 1996; Kerrigan et al., 2000, 2001; Pavol et al., 2002; Cofré et al., 2011; Frimenko et al., 2015). As a result, the coordination was impaired with aging and linked to a history of falls in the past year (Hutin et al., 2011; Chiu and Chou, 2012; Ghanavati et al., 2014; James et al., 2017; Hafer and Boyer, 2018 Hutin et al., 2011; Ghanavati et al., 2014. However, these studies used standard frequency-decomposition methods to evaluate the intersegmental coordination during walking (i.e., continuous relative phase and coding vector). While these methods are well documented, they do not provide a complete overview of the gait processing of the lower limb intersegmental coordination due to their analysis of singular parameters. Indeed, it is known that during human walking, the lower-limb coordination is controlled through a coupling of all the segments (thigh, shank, and foot) in order to simplify the spatiotemporal control of locomotion and equilibrium (Borghese et al., 1996; Lacquaniti et al., 2002). The elevation angles of these segments are consequently related. When lower-limb segment rotations (temporal changes in the elevation angles) are plotted one vs. each others, they covary along a plane and constitute a loop [i.e., *covariation plane*, (Ivanenko et al., 2008; Lacquaniti et al., 2012a)]. Principal component analysis (PCA) was used to analyse that plane and when applied produced three components. In normal walking, the first and the second components define the robustness of the planarity of the loop whereas the third component defines its orientation. In this context, the properties of the covariation plane provide insights about how the central nervous system controls the limbs during walking and therefore might reflect the adaptation of the neural and neuromuscular systems with aging. In particular, the work of Lacquaniti and others (for a details see Ivanenko et al., 2006; Lacquaniti et al., 2012b) postulated that planar covariation may provide a link between neuromuscular control and mechanics of gait by matching the control of lower limb muscle patterns to those of the body's center of mass (Bleyenheuft and Detrembleur, 2012). Consequently, this study aimed to evaluate the impact of aging on the coordination of lower limb kinematics and kinetics during walking at comfortable speed using the planar covariation of elevation angles. We hypothesized that the planar covariation of elevation angles should be modified throughout the lifespan in order to adapt the locomotor pattern to the constraints of aging. To this end, we assessed effects of walking speed and age on the pattern and variability of lower limb intersegmental coordination in a cohort of healthy subjects from 25 to 85 years old.

## Materials and Methods

### Participants

Eighty-four volunteers (51 women and 33 men) from 28 to 85 years old were recruited from a previous asymptomatic cohort (clinical trial registration: NCT02042586) to participate in this prospective study. All showed no symptomatic musculoskeletal, neurological, or cardiovascular disease. Exclusion criteria were significant pain, ankle, hip or foot disorders, chronic back pain, Alzheimer's disease, Parkinson's disease, motor neuron disorders, non-stabilized diabetes mellitus, cardiac or respiratory insufficiency, and any inability to understand the procedures. The study protocol was approved by the local ethics committee (CPP Est I, Dijon, France). The study was conducted in compliance with the principles of Good Clinical Practice and the Declaration of Helsinki, and all patients gave their informed consent.

### Task and Procedure

Participants were asked to walk 10 times barefoot while following a straight-line path, 10 meters in length traced on the floor. After each walking trial, they were asked to return to the starting point. They were instructed to adopt a natural and comfortable gait speed, as if they walked “along the street.” Lower body kinematics (i.e., movements of pelvis, hips, knees, and ankles sagittal, frontal, and transverse plane) during walking were measured using an 8 optoelectronic camera motion capture system (Vicon MX, Vicon®, Oxford, UK) sampling at 100 Hz. The marker set used, the Plug-in-Gait marker set (Davis et al., 1991), was composed of 16 reflective markers positioned on specific anatomical landmarks on the lower limb (see Laroche et al., 2014 for placement on a representative participant).

### Data Analysis

**Figure 1:**
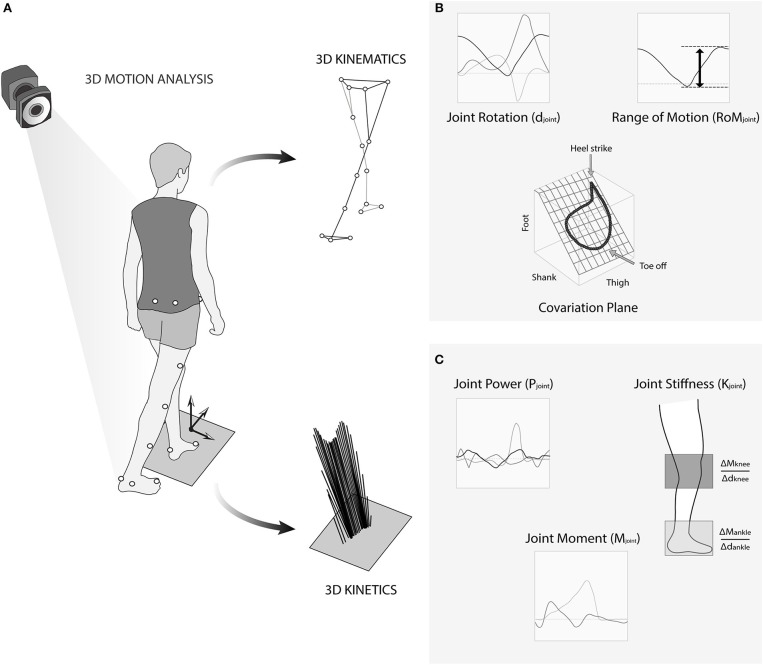
**(A)** Representation of the 3D recording of locomotion with the marker set, with the extracted markers trajectories (Top), and the recorded ground reaction forces (Bottom). **(B)** Details of the joint rotation, joint range of motion, and covariation plane. **(C)** Details of joint power and moment for computing stiffness.

Marker trajectories were recorded by the optoelectronic camera allowing to reconstruct embedded coordinate systems associated to each rigid body segment (pelvis, femur, tibia, and foot) defining then a complete 3-dimensional model of the lower limb. To access kinetics data (i.e., joint moment and power), ground reaction forces were also recorded with two force platforms (AMTI®, USA) sampled at 1,000 Hz (Figure 1A).

Marker trajectories were interpolated with Woltring polynomial and then filtered with a low pass zero phase shift Butterworth filter with a respective cut off frequency of 10 Hz. Similarly, ground reaction forces were filtered with a low pass zero phase shift Butterworth filter with a respective cut off frequency of 50 Hz (van den Bogert and de Koning, 1996). Displacements of the center of mass (CoM), joint kinematics and kinetics were calculated with the Nexus software (Vicon®, Oxford, UK) using inverse dynamic on the Plug-in-Gait model. The gait events were detected using a method proposed by Zeni et al. (2008) and expressed by gait cycle. Briefly, this method defines the heel-strike and the toe-off as the instant where the foot, respectively, begins to move backward and forward in the pelvis frame.

We chose the most representative variables of the gait kinematics, kinetics, and stiffness that could associated with neuromuscular adaptation during gait (Lacquaniti et al., 2012b; Herssens et al., 2018). We first computed amplitude of displacements of CoM in the vertical plane (Amp_CoM_). We then extracted the gait speed (v), step width, step length, and computed the Froude number *Fr* = v^2^/g.L with g as the acceleration due to gravity and L, the subject's leg length. This parameter allows normalizing the velocity across participants (Saibene and Minetti, 2003). We also computed variablility of the step length and step width (Herssens et al., 2018). From the joint kinematics, we computed the range of motion (ROM_Hip_, ROM_Knee_, and ROM_Ankle_) during walking, defined as the sum of the peak flexion and extension, or peak dorsal and plantar flexion (Figure 1B). From the joint kinetics, we extracted the maximal positive power during gait for each joint (P_Hip_, P_Knee_, and P_Ankle_) and computed the associated joint moments normalized by the subject's body weight (M_joint_) (Figure 1C). We then estimated the stiffness of the knee and ankle joints (K_Knee_ and K_Ankle_) using the torsional spring model (Farley and Morgenroth, 1999; Kuitunen et al., 2002). The stiffness (Nm.kg^−1^.deg^−1^) was calculated as a change in the joint moment divided by the change in joint angular displacement in the middle of the ground contact phase (Hobara et al., 2013; Figure 1C).

The spatio-temporal structure of the lower limb intersegmental coordination was evaluated using a principal component analysis. Three segments per lower limb were taken into account: the feet (defined as the virtual lines joining the marker located in the second metatarsal head and the marker located in the lateral malleolus), the shanks (defined as the virtual lines joining the marker located in the lateral malleolus and the marker located in the lateral femoral condyle), and the thighs (defined as the virtual lines joining the marker located in the lateral femoral condyle and the marker located in antero-superior iliac spine). Such analyses were computed randomly for one lower limb independently by means of the covariance matrix of the angular variation of foot, shank, and thigh segments as described previously (Borghese et al., 1996; Bianchi et al., 1998; Lacquaniti et al., 2002; Ornetti et al., 2011). The first two principal eigenvectors, accounting for almost 99% of data variance, correspond to the “covariation plane” (Var_CovPlane_). The temporal coupling between the elevation angles of the shank and the thigh segments (μ_1_) is illustrated with the first eigenvector and its projection on the thigh axis. The temporal coupling between the elevation angles of foot and shank segments were given by the third eigenvector (μ_3_) normal to the plane. All these parameters were obtained for each gait cycle allowing to obtain two values per subject (mean and standard deviation).

### Statistical Analysis

Data analysis was performed with Stata statistical software (version 15.1, Statacorp, College station TX, USA). We first applied univariate correlation between age and either normalized gait speed or planar covariation indices (μ_1_, μ_3_)_._ We applied stepwise regression analysis to identify the most relevant variables associated with age. Entry criterion of the three stepwise procedures was set at 0.20 and stay criterion at 0.10. The procedure stopped when no more variables satisfied the previous criteria. In order to validate the model, the colinearity between variables and the residuals homogeneity were checked, respectively, by the calculation of the Variance Inflation Factor (VIF) and the read of residuals vs. predicted values graphic. A VIF value higher than 5 enabled us to admit colinearity between variables (Kutner et al., 2004), those variables were then removed from the model, if necessary. Data from the gait analysis were entered as follows into the multivariate stepwise linear regression model:

- Kinematics variables (Amp_CoM_, ROM_Hip_, ROM_Knee_, ROM_Ankle_, Var_CovPlane_, μ_1_, μ_3_, step width, Froude Number)
- Kinetics variables (P_Hip_, P_Knee_, and P_Ankle_)
- Stiffness variables (K_Knee_ and K_Ankle_)
- Gait instability parameters (Standard deviation of step length and step width).

Statistical significance was defined as *P* < 0.05. The parameter estimates, 95% confident interval and partial R-square are given and compared to Cohen's suggestions (Cohen, 1992).

## Results

The characteristics of participants are summarized in Table 1.

**Table 1:** Population characteristics.

	**Mean±*SD***
Age (years)	60.7 ± 15.2
Weight (kg)	68.5 ± 13.3
Height (cm)	164.6 ± 9
Gait speed (m.s^−1^)	1.09 ± 0.16
Fr	0.14 ± 0.02
Step Width (m)	0.075 ± 0.02
SD Step Width (m)	0.018 ± 0.01
Step length (m)	0.59 ± 0.06
SD Step length (m)	0.021 ± 0.01
Amp_CoM_ (cm)	2.82 ± 0.52
ROM_Hip_ (°)	40.2 ± 4.56
ROM_Knee_ (°)	55.6 ± 4.52
ROM_Ankle_ (°)	26.1 ± 4.93
P_Hip_ (W)	1.13 ± 0.37
P_Knee_ (W)	0.49 ± 0.21
P_Ankle_ (W)	2.95 ± 0.67
K_Knee_ (Nm.kg^−1^.deg^−1^)	0.07 ± 0.07
K_Ankle_ (Nm.kg^−1^.deg^−1^)	0.06 ± 0.03
Var_CovPlane_ (%)	99.3 ± 0.24
μ_1_	0.44 ± 0.04
μ_3_	-0.16 ± 0.03

*Fr, Froude number corresponding to a normalized self-selected gait speed; Amp_CoM_, amplitude of displacements of the center of mass in the vertical plane; ROM_Hip_, range of motion of the hip in the sagittal plane; ROM_Knee_, range of motion of the knee in the sagittal plane; ROM_Ankle_, range of motion of the ankle in the sagittal plane; P_Hip_, maximal positive hip power; P_Knee_, maximal positive knee power; P_Ankle_, maximal positive knee power; K_Knee_, knee stiffness; K_Ankle_, ankle stiffness; Var_CovPlane_, explained variance of the covariation plane; μ_1_, shank-thigh coordination; μ_3_, foot-shank coordination; SD, Standard Deviation*.

**Figure 2:**
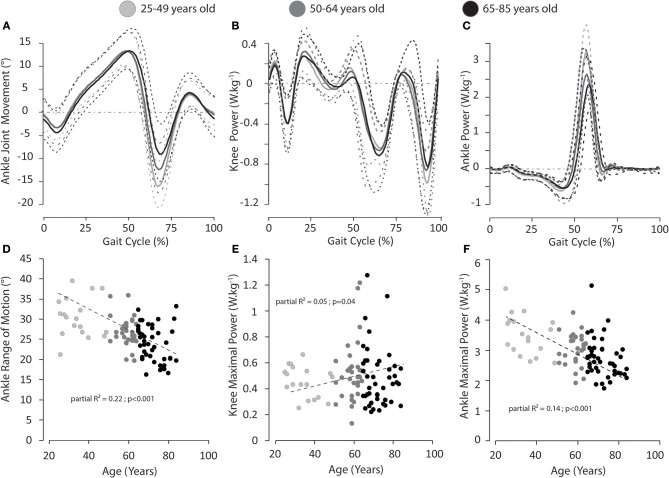
**(A)** Mean (solid lines) and standard deviation (dotted lines) waveforms of sagittal ankle joint excursions for people of 25–49 (light gray)/50–64 (medium gray)/65–85 (dark gray) years old. **(B)** Mean waveforms of knee power for people of 25–49 (light gray)/50–64 (medium gray)/65–85 (dark gray) years old. **(C)** Mean waveforms of sagittal ankle power for people of 25–49 (light gray)/50–64 (medium gray)/65–85 (dark gray) years old. Relationships between age and ankle range of motion **(D)**, knee maximal power **(E)**, ankle maximal power **(F)**. Partial *R*^2^ and *p*-Value are provided. We choose to represent 3 classes of age in order to highlight change due to age.

**Figure 3:**
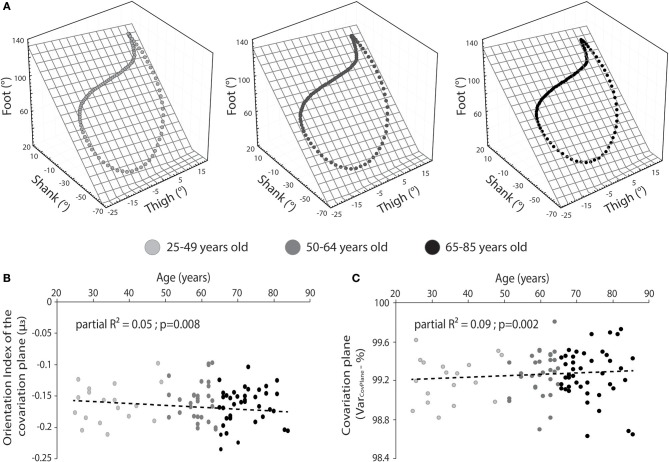
**(A)** Representation of the mean planar covariation of the lower-limb segments for people of 25–49 (light gray)/50–64 (medium gray)/65–85 (dark gray) years old. **(B)** Relationship between the orientation (index) of the covariation plane and the age for people of 25–49 (light gray)/50–64 (medium gray)/65–85 (dark gray) years old. **(C)** Relationship between the variance of the covariance plane and the age for people of 25–49 (light gray)/50–64 (medium gray)/65–85 (dark gray) years old. Partial *R*^2^ is provided. We choose to represent 3 classes of age in order to highlight change due to age.

We performed univariate correlations between normalized self-selected gait speed (Fr) and age and planar covariation (Var_CovPlane_) and age. A negative significant weak correlation (*r* = −0.24; *P* = 0.028) was found between normalized gait speed and age of the participants. Furthermore, we performed a multiple stepwise linear regression analysis between age and parameters computed from gait analysis (see methods for details). The regression model provided a moderate explanation of the variance (*F* = 6.20; adjusted *R*^2^ = 0.49; *p* < 0.001) and revealed no significant relationship between age and normalized gait speed (*p* = 0.11). However, significant relationships were found between age and range of motion of the ankle, maximal power of the knee, and the ankle (Table 2; Figure 2), percentage of planar covariation and the intersegmental foot-shank coordination (Table 2; Figure 3).

**Table 2:** Multivariate linear regression model between kinematics and kinetics variable and age.

**Variables**	**β**	***z***	***p***	**Partial *R*^**2**^**	**95% CI**	**VIF**
ROM_Ankle_	−1.55	−3.98	<0.001	0.22	−2.3; −0.8	2.18
P_Knee_	16.0	2.10	0.04	0.05	1.1; 31	1.37
P_Ankle_	−11.7	−4.13	<0.001	0.14	−17.3; −6.2	1.56
Var_CovPlane_	−23.7	−3.09	0.002	0.09	−38.8; −8.7	1.84
μ_3_	−135.5	−2.65	0.008	0.05	−235.7; −35.4	1.11

*ROM_Ankle_, range of motion of the ankle in the sagittal plane; P_Knee_, maximal positive knee power; P_Ankle_, maximal positive knee power; μ_3_, foot-shank coordination; 95%CI, 95% confidence interval; VIF, variance inflation factor*.

## Discussion

The present study aimed to assess the impact of non-pathological aging on the coordination of lower limb kinematics and kinetics during walking at conformable speed using the planar covariation of elevation angles. We showed the adaptation of planar covariation of lower-limb segments throughout the lifespan and the related kinematics and kinetics during walking.

Our results are first consistent with previous studies that showed a significant effect of aging on gait performance (Boyer et al., 2017), especially walking speed. a significant relationship between aging and normalized gait speed was found which would attest to a decline in speed with age. Interestingly, this relationship was not evident in the multivariate model indicating that confounding variables may have been present. Indeed, while aging is associated with a reduction in gait speed, it has been previously detailed that it also produces a broad range of physiological and biomechanical changes on the walking apparatus, from the loss of muscular strength and mass, to a reduction in joint range of motion (Pavol et al., 2002; Delmonico et al., 2009; Billot et al., 2010; Cattagni et al., 2014). In our study, we corroborate and extend these results by showing that these changes occur specifically at the level of the knee/shank and ankle/foot during walking. Moreover, stiffness at both ankles and knees seems have no influence on joint motion in our results and are in line to those reported by others with no evolution of joint stiffness with age (Ochala et al., 2004; Collins et al., 2018). In the same vein, variability of step length, and step width previously reported as gait instability surrogates did not reach significance in our model. One possible explanation is that the confortable walking velocity might have optimized balance during gait. Further study implementing more complex balance constraints need to explore the contribution of these parameters in the aging process.

Outcomes extracted from lower-limb coordination, ankle motion, and plantar-flexors muscles seem to be a key target for both scientist and therapist. More precisely, ankle power, ankle sagittal kinematic, and shank-foot coordination seem to be reduced with aging whereas knee power seems to increase. Such modifications may reveal a potential adaptive mechanism occurring throughout the lifespan. Consequently, we believe that the planar covariation method provides basic insights into how the central nervous system controls limbs during walking by taking into account the global coordination of the thigh, shank, and foot segments. One can expect that lower-limb coordination has been modified with aging in order to compensate for the weakness progressively shown with aging and especially after the 6th decade of life. Such modifications of lower-limb coordination have been previously reported when the locomotor apparatus is impaired (Laroche et al., 2007; Ornetti et al., 2011; Leurs et al., 2012). However, a previous study (Bleyenheuft and Detrembleur, 2012) failed to observe lower-limb coordination difference with aging. It could be explained by the weak statistical power and the absence of the shank-foot coordination, that seems to be modulated with aging. Thus, the planar covariation method seems to highlight the adaptation of the decline in the neuromuscular system with aging (Lacquaniti et al., 2012b). It could be argued therefore, that lower-limb coordination may act as a compensatory mechanism for physiological, and biomechanical changes in order to optimize the locomotor control and the dynamical balance (Ivanenko et al., 2006). Recently, Song and Geyer (2018) proposed a computer simulation to investigate the physiological causes of altered gait with aging, They found potential evidence that muscle-activation changes dominantly contribute to the reduced walking speed. In others words, the alteration of ankle power with aging could be one of the primary symptoms of the physiological decline due to aging. Further work should investigate muscular activation along lifespan in order to corroborate this hypothesis. A particular attention has to be done on prevention programs specifically designed to enhance the strength and coordination of lower-limb muscles and determine its potential effect of ankle power, lower-limb synergies, and gait speed.

This study does however, have several limitations. First, the power of the multiple regression was limited by the number of volunteers. However, the advantage inherent in this limitation is that only very strong relationships could be demonstrated. Despite the linear relationship between age and walking parameters, this study did not provide longitudinal data of volunteers. However, in the majority of studies, only groups are compared. We provide in this study data from young adults to aging people that may highlights changes during the whole lifetime. Second, the absence of the maximal strength of the volunteers to quantify the functional capacity and possibly the related gait performance should be noted.

In conclusion, this study showed age-related effects on gait performance. In particular, the modification of shank-foot coordination could constitutes a neuromuscular adaptation of the changes (biomechanical, physiological, etc.) occurred with aging. Furthermore, our results might have implications for clinical research and practice. Indeed, these four specific parameters could be relevant outcomes to measure efficacy of rehabilitative strategies and to evaluate their efficiency for restoring lower-limb synergies during walking. Consequently, it may be interesting to focus gait rehabilitation on the improvement of ankle amplitude and power as well as foot-shank coordination with healthy and pathological elderly people.

## Data Availability

The datasets generated for this study are available on request to the corresponding author.

## Ethics Statement

The study protocol was approved by the local ethics committee (CPP Est I, Dijon, France). The study was conducted in compliance with the principles of Good Clinical Practice and the Declaration of Helsinki and all patients gave their informed consent.

## Author Contributions

DL, PO, CL, and CM designed the experiment. DL and PO performed the experiments. MG, DL, AG, J-MC, PS, PO, and CL analyzed the data. MG, DL, and PS drafted the manuscript. MG, DL, PS, AG, J-MC, and PO critical revision of the article for important intellectual content. All authors give final approval of the version to be submitted.

### Conflict of Interest Statement

The authors declare that the research was conducted in the absence of any commercial or financial relationships that could be construed as a potential conflict of interest.

## References

[B1] Bernstein N. A. (1967). The Coordination and Regulation of Movements. Oxford: Pergamon Press.

[B2] Bianchi L. Angelini D. Lacquaniti F. (1998). Individual characteristics of human walking mechanics. Pflugers Arch. Eur. J. Physiol. 436, 343–356. 10.1007/s004240050642 9644215

[B3] Billot M. Simoneau E. M. Van Hoecke J. Martin A. (2010). Age-related relative increases in electromyography activity and torque according to the maximal capacity during upright standing. Eur. J. Appl. Physiol. 109, 669–680. 10.1007/s00421-010-1397-7 20213469

[B4] Bleyenheuft C. Detrembleur C. (2012). Kinematic covariation in pediatric, adult and elderly subjects: is gait control influenced by age? Clin. Biomech. 27, 568–572. 10.1016/j.clinbiomech.2012.01.010 22386536

[B5] Borghese N. A. Bianchi L. Lacquaniti F. (1996). Kinematic determinants of human locomotion. J. Physiol. 494, 863–879. 10.1113/jphysiol.1996.sp021539 8865081 PMC1160684

[B6] Boyer K. A. Johnson R. T. Banks J. J. Jewell C. Hafer J. F. (2017). Systematic review and meta-analysis of gait mechanics in young and older adults. Exp. Gerontol. 95, 63–70. 10.1016/j.exger.2017.05.005 28499954

[B7] Cattagni T. Scaglioni G. Laroche D. Van Hoecke J. Gremeaux V. Martin A. (2014). Ankle muscle strength discriminates fallers from non-fallers. Front. Aging Neurosci. 6:336 10.3389/fnagi.2014.00336 25566068 PMC4271599

[B8] Chiu S.-L. Chou L.-S. (2012). Effect of walking speed on inter-joint coordination differs between young and elderly adults. J. Biomech. 45, 275–280. 10.1016/j.jbiomech.2011.10.028 22078272

[B9] Cofré L. E. Lythgo N. Morgan D. Galea M. P. (2011). Aging modifies joint power and work when gait speeds are matched. Gait Posture 33, 484–489. 10.1016/j.gaitpost.2010.12.030 21256026

[B10] Cohen J. (1992). Statistical power analysis. Curr. Dir. Psychol. Sci. 1, 98–101. 10.1111/1467-8721.ep10768783

[B11] Collins J. D. Arch E. S. Crenshaw J. R. Bernhardt K. A. Khosla S. Amin S. (2018). Net ankle quasi-stiffness is influenced by walking speed but not age for older adult women. Gait Posture 62, 311–316. 10.1016/j.gaitpost.2018.03.031 29609159 PMC5960620

[B12] Davis R. B. Ounpuu S. Tyburski D. Gage J. R. (1991). A gait analysis data collection and reduction technique. Hum. Mov. Sci. 10, 575–587.

[B13] Delmonico M. J. Harris T. B. Visser M. Park S. W. Conroy M. B. Velasquez-Mieyer P.. (2009). Longitudinal study of muscle strength, quality, and adipose tissue infiltration. Am. J. Clin. Nutr. 90, 1579–1585. 10.3945/ajcn.2009.28047 19864405 PMC2777469

[B14] Farley C. T. Morgenroth D. C. (1999). Leg stiffness primarily depends on ankle stiffness during human hopping. J. Biomech. 32, 267–273. 10.1016/S0021-9290(98)00170-5 10093026

[B15] Frimenko R. Goodyear C. Bruening D. (2015). Interactions of sex and aging on spatiotemporal metrics in non-pathological gait: a descriptive meta-analysis. Physiother. 101, 266–272. 10.1016/j.physio.2015.01.003 25702092

[B16] Ghanavati T. Salavati M. Karimi N. Negahban H. Ebrahimi Takamjani I. Mehravar M.. (2014). Intra-limb coordination while walking is affected by cognitive load and walking speed. J. Biomech. 47, 2300–2305. 10.1016/j.jbiomech.2014.04.038 24861632

[B17] Hafer J. F. Boyer K. A. (2018). Age related differences in segment coordination and its variability during gait. Gait Posture 62, 92–98. 10.1016/j.gaitpost.2018.02.021 29544156

[B18] Herssens N. Verbecque E. Hallemans A. Vereeck L. Van Rompaey V. Saeys W. (2018). Do spatiotemporal parameters and gait variability differ across the lifespan of healthy adults? A systematic review. Gait Posture 64, 181–190. 10.1016/j.gaitpost.2018.06.012 29929161

[B19] Hobara H. Inoue K. Kanosue K. (2013). Effect of hopping frequency on bilateral differences in leg stiffness. J. Appl. Biomech. 29, 55–60. 10.1123/jab.29.1.55 23462443

[B20] Hutin E. Pradon D. Barbier F. Gracies J.-M. Bussel B. Roche N. (2011). Lower limb coordination patterns in hemiparetic gait: factors of knee flexion impairment. Clin. Biomech. 26, 304–311. 10.1016/j.clinbiomech.2010.10.007 21074912

[B21] Ivanenko Y. P. d'Avella A. Poppele R. E. Lacquaniti F. (2008). On the origin of planar covariation of elevation angles during human locomotion. J. Neurophysiol. 99, 1890–1898. 10.1152/jn.01308.2007 18272871

[B22] Ivanenko Y. P. Poppele R. E. Lacquaniti F. (2006). Motor control programs and walking. Neuroscientist 12, 339–348. 10.1177/1073858406287987 16840710

[B23] James E. G. Leveille S. G. Hausdorff J. M. Barton B. Cote S. Karabulut M.. (2017). Coordination impairments are associated with falling among older adults. Exp. Aging Res. 43, 430–439. 10.1080/0361073X.2017.1369634 29072539

[B24] Judge J. O. Õunpuu S. Davis R. B. (1996). Effects of age on the biomechanics and physiology of gait. Clin. Geriatr. Med. 12, 659–678. 10.1016/S0749-0690(18)30194-0 8890109

[B25] Kerrigan D. C. Croce U. Della M. M. Riley P. O. (2000). A refined view of the determinants of gait: Significance of heel rise. Arch. Phys. Med. Rehabil. 81, 1077–1080. 10.1053/apmr.2000.6306 10943758

[B26] Kerrigan D. C. Lee L. W. Collins J. J. Riley P. O. Lipsitz L. A. (2001). Reduced hip extension during walking: Healthy elderly and fallers versus young adults. Arch. Phys. Med. Rehabil. 82, 26–30. 10.1053/apmr.2001.18584 11239282

[B27] Kuitunen S. Komi P. V. Kyröläinen H. (2002). Knee and ankle joint stiffness in sprint running. Med. Sci. Sports Exerc. 34, 166–173. 10.1097/00005768-200201000-00025 11782663

[B28] Kutner M. Nachtsheim C. J. Neter J. Li W. (2004). Applied Linear Statistical Models, 5th Edn. Irwin, CA: McGraw-Hillf/Irwin series Operations and decision sciences.

[B29] Lacquaniti F. Grasso R. Zago M. (1999). Motor patterns in walking. News Physiol. Sci. 14, 168–174. 10.1152/physiologyonline.1999.14.4.168 11390844

[B30] Lacquaniti F. Ivanenko Y. P. Zago M. (2002). Kinematic control of walking. Arch. Ital. Biol. 140, 263–272. 10.4449/aib.v140i4.485 12228979

[B31] Lacquaniti F. Ivanenko Y. P. Zago M. (2012a). Development of human locomotion. Curr. Opin. Neurobiol. 22, 822–828. 10.1016/j.conb.2012.03.012 22498713

[B32] Lacquaniti F. Ivanenko Y. P. Zago M. (2012b). Patterned control of human locomotion. J. Physiol. 590, 2186–99. 10.1113/jphysiol.2011.215137 22411012 PMC3424743

[B33] Laroche D. Morisset C. Fortunet C. Gremeaux V. Maillefert J. F. Ornetti P. (2014). Biomechanical effectiveness of a distraction-rotation knee brace in medial knee osteoarthritis: preliminary results. Knee 21, 710–716. 10.1016/j.knee.2014.02.015 24642050

[B34] Laroche D. Ornetti P. Thomas E. Ballay Y. Maillefert J. F. Pozzo T. (2007). Kinematic adaptation of locomotor pattern in rheumatoid arthritis patients with forefoot impairment. Exp. Brain Res. 176, 85–97. 10.1007/s00221-006-0597-1 16915399

[B35] Leurs F. Bengoetxea A. Cebolla A. M. De Saedeleer C. Dan B. Cheron G. (2012). Planar covariation of elevation angles in prosthetic gait. Gait Posture 35, 647–55. 10.1016/j.gaitpost.2011.12.017 22257927

[B36] Lewis C. L. Ferris D. P. (2008). Walking with increased ankle pushoff decreases hip muscle moments. J. Biomech. 41, 2082–2089. 10.1016/j.jbiomech.2008.05.013 18606419 PMC2562040

[B37] Mcgibbon C. A. Krebs D. E. (2001). Age-related changes in lower trunk coordination and energy transfer during gait. J. Neurophysiol. 85, 1923–31. 10.1152/jn.2001.85.5.1923 11353009

[B38] Ochala J. Lambertz D. Pousson M. Goubel F. Van Hoecke J. (2004). Changes in mechanical properties of human plantar flexor muscles in ageing. Exp. Gerontol. 39, 349–358. 10.1016/j.exger.2003.11.004 15036394

[B39] Ornetti P. Laroche D. Morisset C. Beis J. N. Tavernier C. Maillefert J.-F. (2011). Three-dimensional kinematics of the lower limbs in hip osteoarthritis during walking. J. Back Musculoskelet. Rehabil. 24, 201–208. 10.3233/BMR-2011-0295 22142708

[B40] Pavol M. J. Owings T. M. Grabiner M. D. (2002). Body segment inertial parameter estimation for the general population of older adults. J. Biomech. 35, 707–712. 10.1016/S0021-9290(01)00250-0 11955511

[B41] Saibene F. Minetti A. E. (2003). Biomechanical and physiological aspects of legged locomotion in humans. Eur. J. Appl. Physiol. 88, 297–316. 10.1007/s00421-002-0654-9 12527959

[B42] Sepic S. B. Murray M. P. Mollinger L. A. Spurr G. B. Gardner G. M. (1986). Strength and range of motion in the ankle in two age groups of men and women. Am. J. Phys. Med. 65, 75–84. 3963168

[B43] Song S. Geyer H. (2018). Predictive neuromechanical simulations indicate why walking performance declines with ageing. J. Physiol. 7, 1199–1210. 10.1113/JP275166 PMC5878225 29344967

[B44] van den Bogert A. J. de Koning J. J. (1996). On optimal filtering for inverse dynamics analysis, in Proceedings of the IXth Biennial Conference, Canadian Society of Biomechanics (Vancouver, BC: Simon Fraser University), 214–215.

[B45] Winter D. A. Ruder G. K. MacKinnon C. D. (1990). Control of balance of upper body during gait, in Multiple Muscle Systems, eds Winters J. M. Woo S. L. Y. (New York, NY: Springer), 534–41. 10.1007/978-1-4613-9030-5_33

[B46] Zeni J. A. Richards J. G. Higginson J. S. (2008). Two simple methods for determining gait events during treadmill and overground walking using kinematic data. Gait Posture 27, 710–714. 10.1016/j.gaitpost.2007.07.007 17723303 PMC2384115

